# Diagnosis of Idiopathic GHD in Children Based on Response to rhGH Treatment: The Importance of GH Provocative Tests and IGF-1

**DOI:** 10.3389/fendo.2019.00638

**Published:** 2019-09-19

**Authors:** João Soares Felício, Luísa Corrêa Janaú, Marcelle Alves Moraes, Nathalie Abdallah Zahalan, Fabrício de Souza Resende, Manuela Nascimento de Lemos, Norberto Jorge Kzan de Souza Neto, Isabela Imbelloni Farias de Franco, Loyane Tamyres Costa Leitão, Lilian de Souza d'Albuquerque Silva, Maria Clara Neres Iunes de Oliveira, Angélica Leite de Alcântara, Ana Carolina Contente Braga de Souza, Wanderson Maia da Silva, Márcia Costa dos Santos, Natércia Neves Marques de Queiroz, Lorena Vilhena de Moraes, Antônio Bentes de Figueiredo, Ana Luiza Prieto Farinassi, Luciana Marques da Costa Farias, Danielle Dias da Silva, Karem Miléo Felício, João Felício Abrahão Neto

**Affiliations:** ^1^Endocrinology Division, University Hospital João de Barros Barreto, Federal University of Pará, Belém, Brazil; ^2^Department of Medicine, State University of Pará, Belém, Brazil

**Keywords:** IGF-1, IGHD, clonidine test, insulin tolerance test, growth hormone deficiency

## Abstract

**Purpose:** Serum IGF-1 (Insulin like growth factor 1) and Growth Hormone (GH) provocative tests are reasonable tools for screening and diagnosis of idiopathic GH Deficiency (IGHD). However, the average cut-off points applied on these tests have a lower level of evidence and produce large amounts of false results. The aim of this study is to evaluate the sensitivity, specificity, and accuracy of IGF-1 and GH stimulation tests as diagnostic tools for IGHD, using clinical response to recombinant human GH (rhGH) treatment as diagnostic standard [increase of at least 0.3 in height standard deviation (H-SD) in 1 year].

**Methods:** We performed a prospective study with 115 children and adolescents presenting short stature (SS), without secondary SS etiologies such as organic lesions, genetic syndromes, thyroid disorders. They were separated into Group 1 [patients with familial SS or constitutional delay of growth and puberty (CDGP), not treated with rhGH], Group 2 (patients with suspicion of IGHD with clinical response to rhGH treatment), and Group 3 (patients with suspicion of IGHD without growth response to rhGH treatment). Then, they were assessed for diagnostic performance of IGF-1, Insulin Tolerance Test (ITT) and clonidine test (CT) alone and combined at different cut-off points.

**Results:** Based on the ROC curve, the best cut-off points found for IGF-1, ITT, and CT when they were used isolated were −0.492 SDS (sensitivity: 50%; specificity: 53.8%; accuracy: 46.5%), 4.515 μg/L (sensitivity: 75.5%; specificity: 45.5%; accuracy: 52.7%), and 4.095 μg/L (sensitivity: 54.5%; specificity: 52.6%; accuracy: 56.9%), respectively. When we had combined IGF-1 with−2SD as cut-off alongside ITT or CT, we found 7 μg/L as the best cut-off point. In this situation, ITT had sensitivity, specificity and accuracy of 93.9, 81.8, and 90.1%, while CT had 93.2, 68.4, and 85.7%, respectively.

**Conclusion:** Our data suggest that diagnosis of IGHD should be established based on a combination of clinical expertise, auxologic, radiologic, and laboratorial data, using IGF-1 at the −2SD threshold combined, with ITT or CT at the cut-off point of 7 μg/L. Additional studies, similar to ours, are imperative to establish cut-off points based on therapeutic response to rhGH in IGHD, which would be directly related to a better treatment outcome.

## Introduction

Children whose stature is two height standard deviation (H-SD) below the mean for age and sex (1) or who have a height deficit greater than one H-SD relative to the family height should be referred for a complete short stature investigation (2). After considering and excluding other short stature (SS) etiologies such as familial SS (FSS), constitutional delay of growth and puberty (CDGP) and secondary causes (organic lesions, thyroid disorders), the investigation for Growth Hormone Deficiency (GHD) should be conducted (3).

Recently, Hussein et al. (4), evaluating 637 children and adolescents with SS, found FSS in 42% of them, CDGP in 16%, GHD in 12% and idiopathic SS (ISS), aside CDGP and FSS, in 2% (4). Nevertheless, to distinguish the last two conditions, we solely have available provocative GH tests and IGF-1, which present low evidence level and produce large amounts of false results (5, 6) The diagnosis of idiopathic GHD (IGHD) is established by stimulation tests of GH secretion, such as Insulin Tolerance Test (ITT) and the Clonidine Test (CT) (7, 8). Serum IGF-1 has also been used to evaluate the somatotropic axis function.

The current consensus statement and some authors recognizes that a satisfactory therapeutic response to recombinant human GH (rhGH) corresponds to an increase in H-SD of more than 0.3–0.5 after 1 year of treatment, thus confirming the hormonal deficiency (9–11). In addition, increases in predicted height and changes in growth rate are useful to analyze clinical response (12).

Therefore, the aim of this study is to evaluate sensitivity, specificity and accuracy values of IGF-1 and GH stimulation tests as diagnostic tools for IGHD, using clinical response to rhGH as diagnostic standard.

## Methods

### Study Design and Patients

This prospective study aimed to evaluate the diagnostic performance of IGF-1 and provocative GH tests (ITT and CT) at different cut-off points (−1SD and −2SD for IGF-1 and 3, 5, 7, and 10 μg/L for GH). Additionally, it was verified whether the measurement of IGF-1 (at the two cut-off points cited) increases the sensitivity, specificity, and accuracy values of the stimulation tests when used together. We also aimed to define the best cut-off points for GH peaks in the provocative tests for the diagnosis of IGHD with a ROC curve.

Data were collected during medical follow-up and treatment of 115 prepubescent children and adolescents with short stature (patients who had <−2 SD height for age and sex and/or <−1 SD for target height) (1). Other SS etiologies such as organic lesions, genetic syndromes and thyroid disorders were excluded.

Were also excluded from the data analysis: children who entered puberty within the first year of clinical follow-up after starting rhGH treatment and those with low adherence as well as loss of follow-up.

The decision to start rhGH therapy was due to the compliance of at least one of the following clinical criteria: height below −3SD; height between −3SD and −2SD combined with growth rate below percentile 25 for its respective age and sex; or height above −2SD associated with growth rate below −1SD (13). Therefore, the provocative tests were not used to indicate rhGH treatment. Patients with FSS and CDGP were diagnosed based on family history of short stature and auxological criteria along with the presence or absence of bone age delay, indicating the probability of delayed growth and puberty (9, 14).

Subjects were divided into three groups: Group 1 (*n* = 20) (9 patients with FSS and 11 with CDGP diagnosed according to consensus guidelines (9) that were not treated with rhGH), Group 2 (*n* = 62) [IGHD patients with clinical response to rhGH (confirmed diagnosis of IGHD by the increase of at least 0.3 SD in height at the end of a year of treatment with rhGH)], and Group 3 (*n* = 33) (patients who were previously diagnosed as IGHD but with no growth response to treatment with rhGH). After the treatment with rhGH, the last group was considered as having ISS aside CDGP and FSS. In summary, only groups 2 and 3 were treated with rhGH for at least 1 year, between 2010 and 2018. All patients underwent at least one provocative GH test and had normal skull Magnetic Resonance Image (MRI). The study was approved by ethics committee and written informed consent was obtained.

#### Summary of Study Design

In summary, we recruited 115 patients with short stature (SS), without secondary SS etiologies such as organic lesions, genetic syndromes, thyroid disorders, after that, we separated group 1 (*N* = 20), diagnosed with FSS or CDGP based on clinical and auxological criteria. Those patients were not treated with rhGH. The 95 remaining patients were assumed as having IGHD and treated with rhGH in similar range doses for at least 1 year. The decision to treat those patients with rhGH was based on auxological criteria, not by stimulation GH tests. Those patients, just after 1 year, were separated in groups 2 and 3, based on their response to rhGH treatment and considered group 2 (responders) as true IGHD. The group 3 (non-responders) was then, diagnosed as possibly ISS (aside CDGP and FSS) (4). Therefore, we assumed that, because of that, they did not have a so great response as group 2. Just after that, we were looking at results of IGF-1 and GH tests performed, to evaluate their utility to identify those groups before treatment.

### Clinical and Laboratorial Data

The dose of rhGH used by the subjects of the study was 0.7–1 UI/kg/week during the first year of follow-up. Provocative tests used in this study were ITT and CT and were performed after 8-h overnight fasting, starting 30 min after placement of venous catheter with slow saline infusion. Blood samples were collected every 30 min between 0 and 120 min. Insulin was administered intravenously (0.05–0.1 U/kg) and clonidine was administered orally (0.15 mg/m^2^). ITT was considered adequate for somatotropic axis assessment if hypoglycemia of 40 mg/dL or less was reached. All children underwent the second stimulation test on a separate day (at least 1 week apart). None of the subjects performed steroid priming. IGF-I was determined by random serum dosage. Among the 2 groups treated with rhGH, 48 patients underwent both provocative tests, 31 to ITT alone and 16 to CT only.

The following data were also collected from each patient: height, target height, chronological age, bone age, pubertal staging, TSH levels, free T4, FSH, LH, estradiol, total testosterone, IGF-1, and IGFBP-3. Patient's heights were measured in triplicate using the Harpender Stadiometer, as well as the height of their parents. The bone age was based on the analysis of left hand and wrist radiographs, using Greulich and Pyle's standard method (15).

Tanner method was used for pubertal staging (16, 17). Target height was calculated by the Tanner method: (height of the father + height of the mother – 13)/2 for females and (height of the father + height of the mother + 13)/2 for males and expressed in centimeters. Predicted heights were calculated, before and after 1 year of treatment, by the Bayley-Pinneau method (18, 19) based on height and bone age of each patient.

### Assays

GH response to provocative tests (ITT and CT) and serum IGF-I were measured by chemiluminescent immunometric assay (Immulite 1000; Diagnostic Products Corp., Los Angeles, CA, USA). The calibration range for IGF-I was up to 1.6 μg/L against the WHO NIBSC 1st IRR 87/518 and the sensitivity of the test was 20 μg/L. Whether calibration range for GH assay was up to 40 μg/L (WHO 1st IS 80/505 and WHO 2nd IS 98/574) and the sensitivity of the test was up to 0.01 μg/L. Consistency of assay performance was assessed by regular use of internal controls. The GH intra- and inter-assay coefficients of variation were, respectively, 5.3–6.5% at GH levels of 1.7–31 μg/L and 5.5–6.2% at GH levels of 3–18 μg/L. The intra- and inter-assay coefficients of variation for IGF-I were <4.5% and <8.4% (20, 21).

### Statistical Analysis

Data concerning clinical and epidemiological characteristics were processed using descriptive statistic, expressed as Mean ± Standard Deviation, Confidence Interval of 95% and/or as absolute and relative frequencies, as appropriated, and presented in tables and/or graphics.

Paired student's *t*-test for dependent means or Wilcoxon signed-rank test were used to compare variables in each group before-and-after. ANOVA was used to compare variances of variables with normal distribution in more than two groups and Kruskal-Wallis was employed when the variables had non normal distribution. The *p* < 0.05 was considered statistically significant.

The best cut-off point was defined based on Youden Index (J) and, additionally, a ROC (Receiver Operating Characteristic) curve was constructed. The cut-off with maximum sensitivity and specificity in the ROC curve was defined as the minimum value in the equation √ [(1 – sensitivity)^2^ + (1 – specificity)^2^] and the accuracy was estimated based on the area under the ROC curve. Predictive values and likelihood ratios were also calculated from the values of sensitivity and specificity.

H-SDS and PH-SDS were derived from World Health Organization (WHO) charts and tables for growth follow-up (22). Sensitivity, specificity and diagnostic accuracy were expressed as percentage. IGF-1-SDS were derived from Elmlinger et al. (23).

All tests were performed using the SPSS Statistics 22^®^ software (IBM Corp., Armonk, NY, USA). Further, results were considered significant if *p* < 0.05.

## Results

Considering all patients, 84/115 (73%) were male and 31/115 (27%) were female. Age was 9.9 ± 2.7 years. The characteristics of each group are summarized in Tables 1, 2.

**Table 1:** Initial clinical and laboratorial characteristics.

**Characteristics**	**Group 1 (*N* = 20)**	**Group 2 (*N* = 62)**	**Group 3 (*N* = 33)**	***p*-value**
Sex (F/M)	3/17	19/43	9/24	NS
Target height (cm)	161.9 ± 9	165.6 ± 9.6	166.1 ± 7.1	NS
Target height SDS	−1.7 ± 0.9	−1 ± 1.1	−1 ± 0.8	<0.05^*^
Peak GH to ITT(μg/L)	19.4 ± 8.3	3.6 ± 2.6	6.4 ± 8.9	<0.05^†^
Peak GH to CT (μg/L)	18.7 ± 15.9	4 ± 3.1	9 ± 11.8	<0.05^†^

* *p < 0.05 (group 1 vs. other groups)*.

† *p < 0.05 (between all groups)*.

*Group 1: children diagnosed with FSS or CDGP; Group 2: children who gained 0.3 SD in height after 1 year of rhGH treatment; Group 2: children who gained <0.3 SD in height after 1 year of rhGH treatment. F, Female; M, Male; IGF-1, Insulin-like Growth Factor I; ITT, Insulin Tolerance Test; CT, Clonidine Test; NS, Not Significant; FSS, Familial Short Stature; CDGP, Constitutional delay of growth and puberty; rhGH, Recombinant Human Growth Hormone*.

**Table 2:** Clinical and laboratorial characteristics before and after follow-up.

	**Group 1 (*****n*** **=** **20)**		**Group 2 (*****n*** **=** **62)**		**Group 3 (*****n*** **=** **33)**		***p*-value**
	**Initial (Mean ± SD)**	**Final (Mean ± SD)**	**Initial (Mean ± SD)**	**Final (Mean ± SD)**	**Initial (Mean ± SD)**	**Final (Mean ± SD)**	
Age (years)	11.4 ± 2.2	12.5 ± 2.2	9 ± 2.8	10 ± 2.8	10.7 ± 2.1	11.8 ± 2.1	<0.05^*^
BA (years)	8.5 ± 2.6	10.1 ± 2.6	6.7 ± 3.3	8.5 ± 3.4	9.4 ± 2.6	10.8 ± 2.5	<0.05^*^
BMI (kg/m^2^)	15.9 ± 3.1	15.1 ± 3.7	17.3 ± 3.5	17.5 ± 3.3	17.6 ± 3.3	18.5 ± 3.6	NS
BMI-SDS	−1.2 ± 1.7	−1.3 ± 1	0.1 ± 1.7	0.1 ± 1.3	−0.01 ± 1.5	−0.02 ± 1.6	NS
Height (cm)	126 ± 10	131.7 ± 10.6	121.1 ± 17.2	131.2 ± 17.3	127.8 ± 14	135 ± 15.1	<0.05^*^
H-SDS	−3 ± 0.6	−3 ± 0.7	−1.9 ± 1.4	−1.1 ± 1.2	−2.1 ± 1.5	−2.1 ± 1.5	<0.05 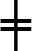 ^†^
PH (cm)	162.3 ± 11	162.1 ± 10	166.5 ± 11.8	172.4 ± 11.9	160.8 ± 13.1	163.2 ± 15.5	<0.05 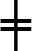
PH-SDS	−1.8 ± 1.4	−1.7 ± 1.3	−0.9 ± 1.1	−0.2 ± 1.2	−1.7 ± 1.4	−1.4 ± 1.8	<0.05 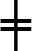
IGFBP3 (μg/mL)	–	–	3.8 ± 1.3	5.2 ± 0.9	4.1 ± 1.4	5.4 ± 0.7	<0.05 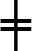 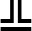
IGF-1 (μg/L)	215.2 ± 100	–	198.6 ± 179	342.1 ± 167.7	194 ± 113	413 ± 160.7	<0.05 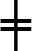 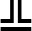
IGF-1 SDS	−0.4 ± 1.5	–	−0.4 ± 2.1	1.5 ± 1.5	−0.7 ± 1.6	0.9 ± 1.4	<0.05 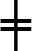 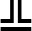
RhGH dose (U/kg/week)	–	–	0.9 ± 0.2	0.9 ± 0.1	0.9 ± 0.1	0.9 ± 0.2	NS

* *p < 0.05 (initial vs. final within all groups)*.

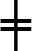
 *p < 0.05 (initial vs. final within group 2)*.

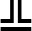
 *p < 0.05 (initial vs. final within group 3)*.

† *p < 0.05 (final vs. final between all three groups)*.

*Group 1: children diagnosed with FSS or CDGP; Group 2: children who gained 0.3 SD in height after 1 year of rhGH treatment; Group 2: children who gained <0.3 SD in height after 1 year of rhGH treatment. BA, Bone age; BMI, Body Mass Index; BMI-SDS, Body Mass Index standard deviations; NS, not significant; H-SDS, Height standard deviations; PH, Predicted Height; PH-SDS, Predicted Height standard deviations; IGF-1, Insulin-like Growth Factor 1; IGF-1 SDS, Insulin-like Growth Factor 1 standard deviations; FSS, Familial Short Stature; CDGP, Constitutional delay of growth and puberty; rhGH, Recombinant Human Growth Hormone*.

The serum GH levels in response to ITT and to CT were different between all groups. When we compared initial and final data of each group, we found a significant increase in H-SD, PH, and PH-SDS only in Group 2 after follow-up. The modifications in BMI were not significant for all three groups. We also found a significant rise in IGF-1, IGF-1 SDS, and IGFBP-3 in groups 2 and 3 (Tables 1, 2).

Comparing only initial data, group 2 differed from groups 1 and 3 for the variables age, bone age and PH-SDS. In addition, group 1 differed from groups 2 and 3 for initial H-SDS and BMI-SDS. When analyzing only final data, the three groups differed for H-SDS and group 2 was different from groups 1 and 3 when we compared PH and PH-SDS. Also group 1 differed from groups 2 and 3 for final BMI-SDS.

The sensitivity, specificity, and accuracy for IGF-1 and GH provocative tests, alone or combined in different cut-off values are shown in Tables 3, 4. Sensitivity for IGHD diagnosis using IGF-1 isolated was 20% for <−2SD and 36% for <−1SD, the specificity was 84.6% and 57.7% for <−2SD and <−1SD, respectively. When we had combined IGF-1 at −2SD cut-off with ITT or CT we found a threshold of 7 μg/L as the best one, with sensitivity, specificity and accuracy of 93.9, 81.8, and 90.1%, and 93.2, 68.4, and 85.7%, respectively.

**Table 3:** Sensitivity, specificity, and accuracy of serum IGF-1 for the diagnosis of IGHD.

**Standard deviation**	**IGF-1**		
	**Sensitivity (%)**	**Specificity (%)**	**Accuracy (%)**
< -2	20	84.6	42.1
< -1	36	57.7	43.4

*IGF-1, Insulin Growth Factor 1; IGHD, Idiopathic Growth Hormone Deficiency*.

**Table 4:** Sensitivity, specificity, and accuracy of GH peaks to GH stimulation tests isolated and in association with IGF-1 SDS for the diagnosis of IGHD.

**CP**		**ITT (%)**	**ITT or IGF-1 <−2SD (%)**	**ITT or IGF-1 <−1SD (%)**	**CT (%)**	**CT or IGF-1 <−2SD (%)**	**CT or IGF-1 <−1SD (%)**	**ITT plus CT (%)**
<3 μg/L	Sens	38.8	42.9	53.1	38.6	45.5	54.6	22.9
	Spec	50	90.9	63.6	63.2	84.2	79	76.9
	Accu	42.3	57.8	56.3	46	57.1	61.9	37.5
<5 μg/L	Sens	81.6	81.6	81.6	70.5	75	79.6	62.9
	Spec	36.4	81.8	54.6	36.8	79	68.4	69.2
	Accu	67.6	81.7	73.2	60.3	76.2	76.2	64.6
<7 μg/L	Sens	93.9	93.9	93.9	93.2	93.2	95.5	88.6
	Spec	22.7	81.8	45.5	26.3	68.4	57.9	53.9
	Accu	71.8	90.1	78.9	73	85.7	84.1	79.2
<10 μg/L	Sens	95.9	95.9	95.9	93.2	93.2	95.5	91.4
	Spec	13.6	81.8	40.9	26.3	68.4	57.9	46.2
	Accu	70.4	91.6	78.9	73	85.7	84.1	79.2

*CP, Cut-off point; GH, Growth Hormone; IGF-1 SDS, Insulin-like Growth Factor 1 Standard Deviations; IGF-1, Insulin-like Growth Factor 1; IGHD, Idiopathic Growth Hormone Deficiency; ITT, Insulin Tolerance Test; CT, Clonidine Test; Sens, sensitivity; Spec, specificity; Accu, accuracy*.

**Figure 1:**
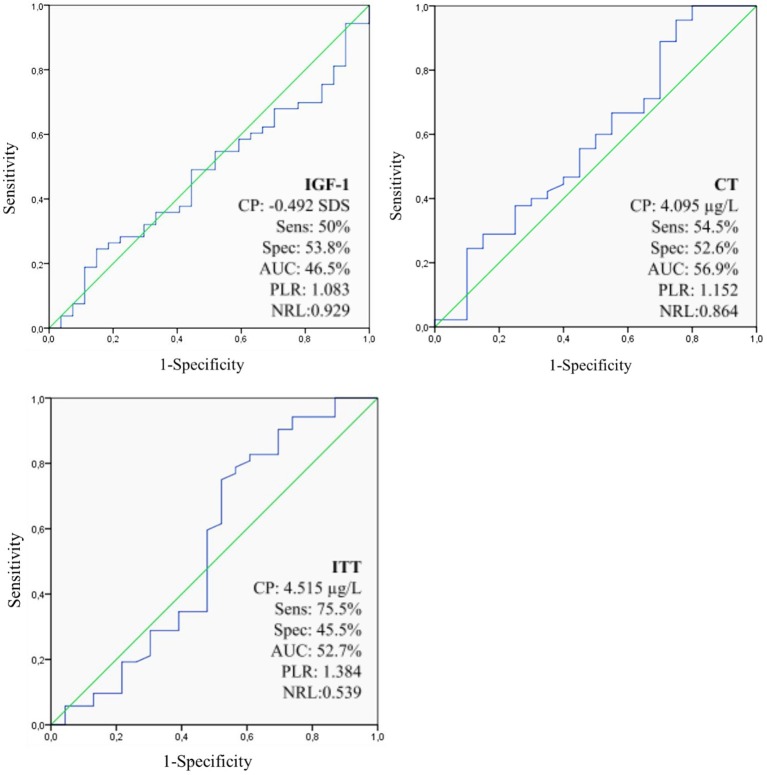
Sensitivity, specificity, accuracy, PLR, and NLR optimal cut-off points for GH response to GH stimulation tests and IGF-1 on the diagnosis of IGHD based on ROC curve approach. IGF-1, Insulin-like Growth Factor 1; CP, Cut-off point; SDS, Standard Deviations; CT, Clonidine Test; AUC, Area Under the ROC curve; ITT, Insulin Tolerance Test; IGHD, Idiopathic Growth Hormone Deficiency; GH, Growth Hormone; PLR, Positive Likelihood Ratio; NLR, negative likelihood ratio; Sens, sensitivity; Spec, specificity.

Based on ROC curve, the best cut-off points for IGF-1, ITT, and CT were −0.492 SDS, 4.515 and 4.095 μg/L, respectively (Figure 1).

## Discussion

Our data suggests the diagnosis of IGHD should be established based on a combination of clinical expertise, auxologic, radiologic, and laboratorial data, using IGF-1 at the −2SD threshold combined with ITT or CT at the cut-off point of 7 μg/L. As we are aware, our study is the first to establish optimal ITT, CT, and IGF-1 cut-off points to identify patients with IGHD using rhGH therapeutic response as diagnostic standard.

There are lots of limitations to detect IGHD in children. Cut-off values of GH peaks described in literature are controversial, ranging from 3 to 10 μg/L (24–26). The major problem in establishing the optimal cut-off point is the lack of a gold standard for GHD diagnosis and the overlapping of results in normal children (27). To deal with this, we used H-SD gain after 1 year of treatment with rhGH to confirm diagnosis (9, 28, 29).

A review by Paula and Czepielewski recommended that GHD should be confirmed by two GH stimulation tests with response lower than 5 μg/L (30). Guzzetti et al. (31), in a study with 74 patients with organic GHD, found 5.1 and 6.8 μg/L as cut-off values for ITT and CT, respectively (31). In addition, Wagner et al. (32) found a 7.09 mcg/L threshold. None of the authors considered as diagnostic standard the therapeutic response to rhGH, using a set of clinical and laboratory variables or radiological modifications. In fact, in our study, the provocative GH tests showed low specificity in all thresholds when used alone and it poorly improved when we combined both, CT and ITT. The best specificity was found when we combined IGF-1 with at least one provocative test, with the threshold of 7 mcg/L. This finding is also aligned with the current European trend that the ideal cut-off among the traditional ones should be near 7 μg/L for modern methods and references, which contradicts the tendency adopted in the 1990 decade, when most physicians rather arbitrarily accepted 10 μg/L as main threshold (9, 33, 34).

When assessed isolatedly, the diagnostic performance of both provocative tests are equivalent in all traditional cut-off points. However, when combined with IGF-1 at the −2 SD cut-off point, ITT showed higher specificity in the 5, 7, and 10 μg/L thresholds when compared to CT, suggesting that the combination of ITT and IGF-1 would be a better choice for IGHD diagnosis.

Levels of IGF-1 alone presented low accuracy for the diagnosis of GHD, with the cut-off point −2SD showing the best results due to higher specificity (84.6%), since it is a more relevant parameter to diagnose low prevalence illnesses (35). Our results are in accordance with a meta-analysis performed by Shen et al. (36), with 12 studies and 1,762 subjects, who reported a specificity of 69% for IGF-1 in the diagnosis of GHD, when using the −2SD as cut-off (36). Many studies recommend that IGF-1 isolated cannot be used to confirm GHD, however it should be applied with the stimulation tests as a complementary tool (37–39). In addition, some authors suggest that IGF-1 should be used, along with auxologic parameters, as screening test for IGHD and that provocative tests should only be performed as a next step in the investigation if serum levels of this exam are low (40, 41). In our study, IGF-1 alone showed very low sensitivity, but we have reached reasonable accuracy performing IGF-1 plus at least one provocative test (ITT or CT) as first approach to diagnose IGHD, after excluding other SS causes. In addition, based on the ROC curve approach, our study showed that the best cut-off point for IGF-1 alone would be −0.492 SDS (sensitivity: 50%, specificity: 53.8%, accuracy: 46.5%). Our ROC curve data showed that all tests (ITT, IGF-1, and CT) presented poorly results when used isolatedly. Therefore, analyzing possible test combinations to boost all diagnostic parameters, we found −2SD for IGF1 as the best cut-off point when associated with both ITT and CT to identify IGHD patients. When IGF1 was used combined with ITT or CT, it keeps high specificity and increases sensitivity and accuracy dramatically. Although, we point out that the ROC curve was just complementary data. The main study results are summarized in Tables 2, 4.

IGF-1 and IGFBP-3 levels increased in both groups 2 and 3 during rhGH treatment. There was no difference in both measurements at the beginning of the study, even though GHD patients had shown relatively low IGFBP-3 levels for age and sex (23). In fact, it has been described that treatment with rhGH has resulted in elevation of both IGF-1 and IGFBP-3 levels in GHD and non-GHD patients and the most pronounced increases were observed 3 and 12 months after treatment started, but not later (42). In addition, there is not a clear relationship between height velocity, GH dose, and circulating IGF-1 and IGFBP-3 levels during GH treatment. In other words, GH/IGF-1/IGFBP-3 system cannot be assessed exclusively by blood levels (9, 11, 42). Finally, in our study, the increase in IGF-1 and IGFBP-3 levels cannot be used to distinguish good responders (group 2) and poor responders (group 3).

In group 3, all subjects were diagnosed with Idiopathic Short Stature (ISS), aside CDGP and FSS, due to not filling the criteria of increasing at least 0.3 SD in height after a 1-year treatment with rhGH. ISS has a variety of causes associated to GH secretion disorders in combination with genetic factors that influence growth physiology. Therefore, for proper diagnosis, are considered a H-SD lower than −2SD for age and sex in addition to a subnormal growth rate, delayed bone age, no apparent medical cause for growth failure (brain injury history, systemic, endocrine, nutritional, and chromosomal abnormalities or being born small to gestational age), and normal growth hormone (GH) response to provocative testing (43). Children with ISS are of normal size at birth but grow slowly during early childhood, so height is within the range for ISS at school beginning (44, 45). In 2003, the U.S. Food and Drug Administration approves the rhGH as a treatment for ISS and several studies have reported positive results in that approach (46–48). However, the height gain seems to be dose-dependent, obtained in those receiving higher dose as reported by Albertsson-Wikland et al. (49). In this scenario, it becomes more imperative to discriminate these patients from IGHD as early as possible, to adequate the rhGH dose and reach a better final height.

The main limitation of the present study was not to perform priming in prepubertal boys older than 11 years and in prepubertal girls older than 10 years (7). Although several studies indicate administration of sex steroid priming, as in Marin et al. (50), there is still controversy about its use (11, 51). Also, there is no consensus about age of administration, type, dose or precise schedule for sex steroid priming during GH stimulation tests (52) as shown in a survey with members of the European Society for Pediatric Endocrinology that used sex steroid priming in 51% of boys and 41% of girls, demonstrating lack of consensus between specialists (53). For these reasons, the decision to prime with sex steroids is country dependent.

The study main strengths are: large number of subjects, consistent response to the treatment with rhGH, IGF-1 data and statistical combination of results, as well as being the first paper, as far as we are aware, to use therapeutic response as diagnostic standard to confirm the IGHD diagnosis. Additionally, our groups were composed only by SS cases whose diagnosis is harder, once there were no brain radiological findings.

Thus, stimulation tests remain reasonable tools, when associated with clinical evaluation, to diagnose children with GHD (11), despite being far from ideal (54). The data of the present survey confirms that the cut-off point for GH peak used in researches and clinical practice needs to be standardized. Seeking for efficiency and uniformity, our study presented the differential of being the first to use SD of height gain in the first year of treatment with rhGH as a parameter to confirm diagnosis of IGHD.

## Conclusion

Our data suggest that diagnosis of IGHD should be established based on a combination of clinical expertise, auxologic, radiologic, and laboratorial data, using IGF-1 at the −2SD threshold combined with ITT or CT at the cut-off point of 7 μg/L. Additional studies, similar to ours, be imperative to establish cut-off points based on therapeutic response to rhGH in IGHD, which would be directly related to a better treatment outcome.

## Data Availability Statement

The datasets analyzed during the current study were available from the corresponding author on reasonable request.

## Ethics Statement

All procedures followed were in accordance with the ethical standards of the responsible committee on human experimentation (institutional and national) and with the Helsinki Declaration of 1975, as revised in 2008. This study was approved by University Hospital Joao de Barros Barreto ethics committee. This manuscript has not been published and is not under consideration for publication in any other journal. All authors approved the manuscript and consent to this submission. Informed consent was obtained from all patients for being included in the study.

## Author Contributions

KF and JF took part in conception and design of study. LJ, MM, NQ, AFi, AFa, LM, AA, DS, and AC were responsible for acquisition of data, while LJ, JF, NZ, FS, NS, WS, and JA have done the analysis and interpretation of data. ML, MS, LL, LS, IF, LF, and MO have drafted the manuscript together. All authors have revised the manuscript critically and approved the version to be published. All persons who meet authorship criteria are listed as authors, and all authors certify that they have participated sufficiently in the work to take public responsibility for the content, including participation in the concept, design, analysis, writing, or revision of the manuscript.

### Conflict of Interest

The authors declare that the research was conducted in the absence of any commercial or financial relationships that could be construed as a potential conflict of interest.

## Abbreviations

AUC: Area Under the Curve
BMI: Body Mass Index
CDGP: Constitutional delay of growth and puberty
CT: Clonidine test
CP: Cut-off point
FSH: Follicle stimulating hormone
FSS: Familial short stature
GH: Growth Hormone
GHD: Growth Hormone Deficiency
H-SDS: Height standard deviations
HUJBB: Hospital Universitário João de Barros Barreto
IGHD: Idiopathic Growth Hormone Deficiency
IGF-1: Insulin Growth Factor 1
IGFBP-3: Insulin Growth Factor Biding Protein 3
J: Youden Index
ITT: Insulin tolerance test
LH: Luteinising Hormone
LR+: Positive Likelihood Ratio
LR–: Negative Likelihood Ratio
MRI: Magnetic Resonance Image
NPV: Negative Predictive Value
PPV: Positive Predictive Value
PH-SDS: Predicted Height standard deviations
RhGH: Recombinant Human Growth Hormone
ROC: Receiver Operating Characteristic
SD: Standard Deviation
TSH: Thyroid Stimulating Hormone.
